# Active exterior cloaking of an inclusion with evanescent multipole devices for flexural waves in thin plates

**DOI:** 10.1098/rsta.2022.0072

**Published:** 2022-11-28

**Authors:** F. J. P. Allison, Ö. Selsil, S. G. Haslinger

**Affiliations:** Department of Mathematical Sciences, University of Liverpool, Liverpool L69 7ZL, UK

**Keywords:** active cloaking, evanescent devices, flexural waves, cloaking, elastic plate, multipole devices

## Abstract

We present an active exterior cloak for flexural waves propagating in a Kirchhoff plate of infinite extent. The evanescent multipole devices are characterized by Macdonald functions $K_{n}$ of the required order, which, assuming time-harmonic vibrations, are solutions of the fourth-order biharmonic equation. It is shown that in the region of interfering waves, which emanate from the devices, a field is recreated which cancels the incident wave to yield a region of ‘stillness’. An inclusion is then positioned in this region for further investigation, with additional attention given to the boundary condition.

This article is part of the theme issue ‘Wave generation and transmission in multi-scale complex media and structured metamaterials (part 2)’.

## 1. Introduction

Thousands of articles have been published since the groundwork of the science of optical illusion was laid. Transformational optics began with Dolin [1], who demonstrated the form-invariance of the Maxwell equations under a space-deforming transformation and proposed a physical application whereby a plane wave passes through a spherical material inhomogeneity without distortion (also see [2]). Much more recently, transformational cloaking was re-established byGreenleaf *et al.* [3], who studied the invariant form of the conductivity equations under coordinate transformations, and thereafter the invariance of the Maxwell equations was investigated independently by Leonhardt [4] and Pendry *et al.* [5], both articles showing how to guide electromagnetic waves around a region to be cloaked. Other contemporaneous studies include the work of Alù & Engheta [6], which presented a design of lossless metamaterial coatings to drastically reduce the scattering cross-section of spherical and cylindrical objects, making them nearly invisible. However, it is well known that the idea of neutral inclusions goes back to Mansfield [7], who examined how to make certain reinforced holes without altering the stress distribution in a uniformly stressed plate. Similar ideas were soon broadened from electromagnetic waves to sound waves, water waves and elastic waves, to count a few. More recently, passive cloaks that surround the object to be hidden from the incoming wave were replaced by active ones which adjust to different frequencies, the shape of the inclusion to be cloaked and the boundary conditions posed on the inclusion itself. In Milton and his co-workers’ seminal work [8–10], an active exterior cloak, where the cloaking region lies outside the cloaking devices (in contrast to an interior cloak, where a cloaking device surrounds the object to be cloaked), was introduced for waves governed by the two-dimensional Laplace and Helmholtz equations. The generalization of their novel idea to three dimensions followed shortly after in [11], and their explorations for the dynamic case were subsequently complemented by Norris *et al.* in [12], where analytical expressions for the device amplitude coefficients for general incidence were given.

It was recently shown by O’Neill *et al.* in [13] that a small number of active monopole sources were sufficient to cloak a clamped inclusion in a Kirchhoff plate. A different approach, based on [8–10], using multipole devices was devised by Futhazar *et al.* [14] to create a finite ‘still’ region and to ensure that only the incident field was present in the far field. O’Neill *et al.* later extended their investigation to the cloaking of coated inclusions in thin plates for frequency ranges in which scattering resonances occur [15] and to the cloaking of finite clusters of pins in thin plates [16].

**Figure 1.**
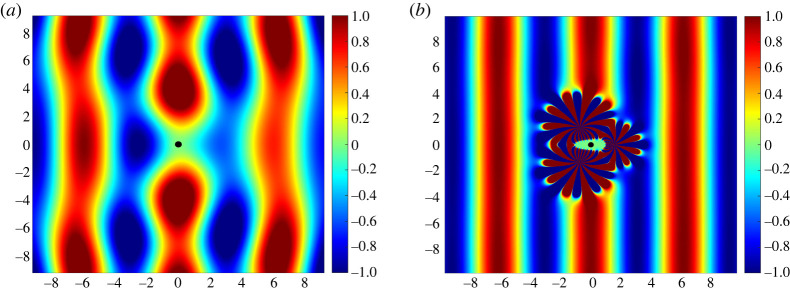
(*a*) The total field $u_{\text{tot}}$ obtained for a plane wave incident on a clamped inclusion. (*b*) The total field after the introduction of three active evanescent devices creating an effective cloak and a finite region of ‘stillness’ surrounding the inclusion, represented by the green zone. (Online version in colour.)

Here, we present the idea of using active evanescent multipole devices to cloak and ‘shield’ an inclusion in a thin plate. In particular, we make use of the fact that the solution to the time-harmonic biharmonic equation consists of not only Hankel functions of the first kind, $H_{n}^{\left(1\right)}$, but also Macdonald functions $K_{n}$ (see [17]). By shielding, we convey a finite region of ‘stillness’ in the vicinity of the inclusion. The reason we use the quotation marks is to emphasize, here and in what follows, the approximate nature of the stillness (or silence for acoustics) of these regions. It should be noted that, although the Green’s function for the biharmonic equation is in fact bounded, we use solely the Macdonald functions to construct our elastic cloak and therefore consent to infinite fields at the device centres, since $K_{0}(z)∼\text{log}(z)$ and $K_{n}(z)∼1/z^{n}$ as z→0. Unlike the Hankel functions, however (used as a basis to formulate the acoustic exterior cloak in [8–10]), the fields emanating from the evanescent devices decay at infinity and thus require less attention than their acoustic analogue. It is known that clamped inclusions in thin plates possess the most scattering signature, as illustrated in figure 1*a*. We preview our results with the use of three active evanescent devices in figure 1*b*.

The structure of the article is as follows. In §2, we formulate the problem for an inclusion in an infinite Kirchhoff plate and present the general solution. Section 3 introduces the evanescent multipole devices of which three are initially used to create a region of ‘stillness’ in our first simulation. Next, we employ a second simulation to compare our previous results with the outcome of a configuration of two devices. We conclude that the former is preferable, although the latter may deserve more attention in the future. The study of cloaking and shielding a circular inclusion is presented in §4. Finally, we conclude and briefly discuss future plans in §5.

## 2. Problem formulation

The out-of-plane elastic displacement W(x;t) in an infinite Kirchhoff plate satisfies the equation of motion

$$D\nabla^{4}W+\rho h\frac{\partial^{2}W}{\partial t^{2}}=0,\;\text{x}\in R^{2},\;t\in (0,\infty ),$$

where $\text{x}=(x_{1},x_{2})$, $\nabla^{4}=\Delta^{2}$ is the biharmonic operator, ρ,h and $D=Eh^{3}/[12(1-\nu^{2})]$ are the mass density, thickness of the plate and plate’s flexural rigidity, respectively, with E and ν representing Young’s modulus and Poisson’s ratio of the elastic material. Assuming time-harmonic vibrations, W(x;t)=w(x)exp⁡(iωt), the governing equation above may be reduced to

2.1 $$(\nabla^{4}-\beta^{4})w=(\nabla^{2}+\beta^{2})(\nabla^{2}-\beta^{2})w=0,\;\text{x}\in R^{2},$$

where $\beta^{2}=\omega \sqrt{\rho h/D}$ is the spectral parameter. The general solution of (2.1) is a linear combination of solutions to the Helmholtz and modified Helmholtz equations, and may be written in polar coordinates r and θ as

2.2 $$w=\sum_{n=-\infty }^{\infty }[A_{n}J_{n}(\beta r)+E_{n}H_{n}^{\left(1\right)}(\beta r)+B_{n}I_{n}(\beta r)+F_{n}K_{n}(\beta r)]\;e^{in\theta },$$

where $J_{n}$ are the Bessel functions of the first kind, $H_{n}^{\left(1\right)}$ are the Hankel functions, and $I_{n}$ and $K_{n}$ are the modified Bessel functions of the first kind and the Macdonald functions of order *n*, respectively. In the presence of an inclusion D, we propose to consider clamped boundary conditions, that is,

2.3 $$w=\frac{\partial w}{\partial \text{n}}=0,\;\text{x}\in \partial D.$$

Here, n is the unit outward normal. In this case, equation (2.1) is satisfied for $\text{x}\in R^{2}\setminus \bar{D}$, where $\bar{D}$ denotes the inclusion together with its boundary.

## 3. Evanescent multipole devices

The objective is to cultivate a region of ‘stillness’ in the vicinity of the origin of the Kirchhoff plate where the incident wave does not propagate; in theory, within this region, an inclusion may be situated without inducing a scattered field. We follow the footsteps of Milton and his co-workers in their ground-breaking work [8–10]; however, as discussed in the problem formulation, here we are dealing with the fourth-order biharmonic equation instead of the second-order Helmholtz equation. The presence of the modified Bessel functions in the solution of the biharmonic equation provides us with additional flexibility. By choosing only the Macdonald functions, $K_{n}$, as the basis for the multi-polar sources and neglecting the Hankel functions, we construct the so-called evanescent multipole devices. As a result, the analogous treatment of the active exterior cloak for a thin plate somewhat simplifies. In fact, we only require that the devices cancel the incident wave $u_{\text{inc}}$ inside a small closed region Ω surrounding the origin, since the constraint of decay at infinity is automatically satisfied by the nature of the evanescent devices.

We denote the sum of the fields generated by Q evanescent devices located at a distance $|{\text{x}}_{s}^{\left(q\right)}|=\delta$ from the origin (outside the region Ω) by $u_{\text{dev}}(r,\theta )$ and require that $u_{\text{dev}}=-u_{\text{inc}}$ for r∈Ω. The desired field may be actualized by taking a linear combination of outgoing waves emanating from the devices’ centres ${\text{x}}_{s}^{1},\ldots ,{\text{x}}_{s}^{Q}$ as

3.1 $$u_{\text{dev}}=\sum_{q=1}^{Q}\sum_{n=-\infty }^{\infty }B_{q,n}K_{n}(\beta r^{\left(q\right)})\;e^{in\theta^{\left(q\right)}},$$

where $B_{q,n}$ are the amplitudes associated with the qth device of nth multipole order, $r^{\left(q\right)}=|\text{x}-{\text{x}}_{s}^{\left(q\right)}|$ and $\theta^{\left(q\right)}=\text{arg}(r^{\left(q\right)})$. We truncate the infinite summation from −N to N and denote the total number of poles in use by M=2N+1. In order to determine the coefficients $B_{q,n}$ numerically, as in [8,9], we first discretize the boundary of Ω into P points ${\text{p}}_{1},\ldots ,{\text{p}}_{P}$. The resulting linear equations are $\text{Ab}\approx -{\text{u}}_{\text{inc}}$, where $\text{b}\in C^{QM}$ is a vector with the coefficients $B_{q,n}$, the matrix $\text{A}\in C^{P\times QM}$ is constructed so that ${\left(\text{Ab}\right)}_{k}=u_{\text{dev}}({\text{p}}_{k})$ and $-{\text{u}}_{\text{inc}}\in C^{P}$ is built by using discrete values of a given incident field, and as such ${\left({\text{u}}_{\text{inc}}\right)}_{k}=u_{\text{inc}}({\text{p}}_{k})$. In fact, $u_{\text{inc}}$ is chosen to be an incident plane wave defined explicitly and in terms of its Jacobi–Anger expansion as

3.2 $$u_{\text{inc}}=e^{i\beta r\cos\theta }=\sum_{n=-\infty }^{\infty }i^{n}J_{n}(\beta r)\;e^{in\theta }.$$

As we work with the fourth-order biharmonic equation, it is necessary to note that the normal derivative of the total field on the boundary of Ω must be small, and this has been verified numerically. Also note that, unlike in [8,9], a condition imposed sufficiently far away from the devices to uphold the decay of the radiating fields is not necessary.

### (a) Simulations

**Figure 2.**
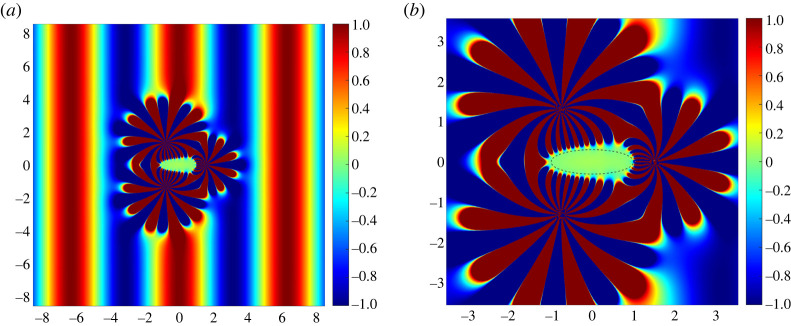
(*a*) The real part of the field $u_{\text{com}}=u_{\text{dev}}+u_{\text{inc}}$ with three devices. (*b*) A close-up of (*a*) with the superimposed dashed black line indicating the boundary of the elliptical region we discretize and on which we impose the condition $u_{\text{dev}}({\text{p}}_{k})\approx -u_{\text{inc}}({\text{p}}_{k})$. The parameters are β=1, Q=3, N=15 and δ=1.5. (Online version in colour.)

**Figure 3.**
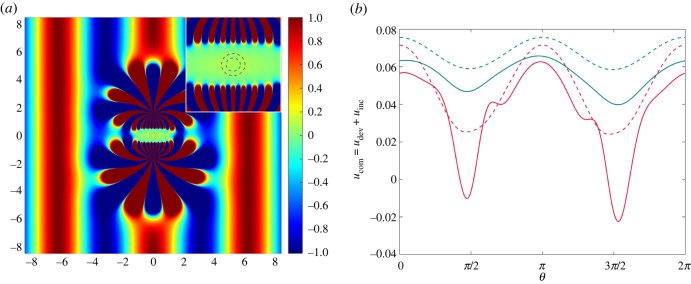
(*a*) The real part of the field $u_{\text{com}}=u_{\text{dev}}+u_{\text{inc}}$ generated by two devices positioned with up–down symmetry. The blue and red dashed concentric circles of radii 0.15 and 0.25, respectively, have been magnified and superimposed for clarity. (*b*) A plot of the displacements measured on the perimeter of the circles. The colour-coded dashed lines correspond to the dashed circles in (*a*) and the solid lines represent the field measured on identical concentric circles inside the ‘still’ region of figure 2. The parameter values are exactly the same as in figure 2. (Online version in colour.)

An overdetermined system of equations with P>QM may be solved, in the least-squares sense, using the singular value decomposition (SVD) method (see, for example, [18], ch. 12). Using SVD we may rewrite the coefficient matrix as $\text{A}=\text{U}\Sigma {\text{V}}^{†}$, where the superscript † denotes the Hermitian transpose. Here, U and V are unitary matrices and Σ is a rectangular diagonal matrix, the elements of which are the singular values of A. Multiplying through by the Moore–Penrose inverse ${\text{A}}^{+}=\text{V}\Sigma^{-1}{\text{U}}^{†}$, we solve for an optimal choice of coefficients for given parameters δ, β and M and the geometrical dimensions of Ω. In figure 2*a*, we illustrate the combined field $u_{\text{com}}=u_{\text{dev}}+u_{\text{inc}}$ (see (3.1) and (3.2)) for β=1, with the device centres distributed evenly on a circle of radius δ=1.5, and its close-up is shown in figure 2*b*. For all the figures in this section, the cloaked region Ω is chosen to be elliptical with minor and major axes 0.3 and 1, respectively, and with its boundary discretized into P=200 points. For these given parameters, we observe that assigning M=31 unique amplitudes to each device yields the optimum silent region; introducing additional poles requires re-evaluation of the scaling of the problem. Note that it is possible to observe from figure 2*a* that the evanescent cloak perturbs only the local fields while leaving the propagating far field intact. The green zone at the omphalos of the figure is a ‘still’ region where the plate is immune from the effects of the incident wave and therefore a haven to cloak an object from detection. In [8–10], it has been proven that the configuration must consist of at least three devices to act as an exterior cloak, the silent region of which ‘is the complement of the union of the three discs’ [10]. However, we experiment with the idea of using fewer than three devices, and in figure 3*a* we present a cloaking attempt with the same parameters as in figure 2*a* but for two, rather than three, devices.

In figure 3*a*, two devices with up–down symmetry visibly create a region of ‘stillness’ that looks comparable to that in the three-device set-up. Quantitatively, we may depict the displacement inside the cloaked region by measuring the field $u_{\text{com}}=u_{\text{dev}}+u_{\text{inc}}$ on two concentric circles, purposefully zoomed in and superimposed on figure 3*a* (see the inset). The dashed lines in figure 3*b* pertain to the two-device configuration in figure 3*a* and indicate that the field becomes larger towards the centre of the region of ‘stillness’. We attribute this growth to the evanescent nature of the cloak, in the sense that $u_{\text{dev}}$ captures $-u_{\text{inc}}$ less well the further it propagates into the epicentre, interestingly exposing the advantage, decay at infinity, and the disadvantage, reconstructing the field $-u_{\text{inc}}$ across a larger region, of the evanescent cloak. In figure 3*b*, we also plot the solid lines which represent the field measured on identical concentric circles inside the ‘still’ region of figure 2. It is clear that, although the displacement generated by the two devices is small, the three-device configuration provides better protection from the incident wave. Whether the scattering from an inclusion positioned at the origin due to the remnant field inside the ‘still’ region is negligible or not is discussed in the next section.

## 4. Cloaking of a clamped inclusion with evanescent multipole devices

In this section, we introduce an inclusion to the region of ‘stillness’ and demonstrate that the active evanescent devices are effective tools for cloaking even a clamped inclusion in a Kirchhoff plate. In contrast to this, as shown in [19], such a task proves impossible for a passive cloak for flexural waves. We also note that in [14] an active cloak for flexural waves in a thin plate was constructed using four devices with large multipole expansions, and a reduction in these expansions had to be compensated for by an increase in the number of devices themselves. Moreover, the efficacy of the devices in cloaking an inclusion in the ‘still’ region was left uninvestigated, despite this being an important aspect. A successful cloaking technique based on a small number of active monopoles in a thin plate was presented in [13]. For the sake of convenience, let us assume that the inclusion we would like to cloak is a circular disc of radius $r_{d}$. In agreement with the notation in §2, we let w denote the sum of the combined field $u_{\text{com}}=u_{\text{dev}}+u_{\text{inc}}$ in the ‘still’ region and the resulting scattering $u_{\text{sca}}$ due to $u_{\text{com}}$. As the disc is clamped, we simply refer to the boundary conditions (2.3) and note that the normal derivative is now in the radial direction. We are thus left with

4.1 $$-u_{\text{com}}(r_{d})=u_{\text{sca}}(r_{d})\;\mathrm{and}\;-u_{\text{com}}^{\prime }(r_{d})=u_{\text{sca}}^{\prime }(r_{d}),$$

where prime ${\;}^{\prime }$ denotes the derivative in the radial direction. The left-hand sides of these two equations are determined numerically and approximated by respective complex Fourier series, the coefficients of which are then used to solve for outgoing wave coefficients $E_{n}\text{ and }F_{n}$ (see (2.2)), as the scattered field $u_{\text{sca}}$ is explicitly given by

4.2 $$u_{\text{sca}}=\sum_{n=-\infty }^{\infty }[E_{n}H_{n}^{\left(1\right)}(\beta r)+F_{n}K_{n}(\beta r)]\;e^{in\theta }.$$

Comparing the coefficients and retaining the above series for n from −N to N, a matrix equation Ks=c is constructed where (with prime ${\;}^{\prime }$ denoting derivative with respect to the argument)

$$\text{K}=\left[\begin{array}{cccccccc} H_{-N}^{\left(1\right)}(\beta r_{d}) & 0 & \ldots & 0 & K_{-N}(\beta r_{d}) & 0 & \ldots & 0\\ 0 & \ddots & & \vdots & 0 & \ddots & & \vdots \\ \vdots & & \ddots & 0 & \vdots & & \ddots & 0\\ 0 & \ldots & 0 & H_{N}^{\left(1\right)}(\beta r_{d}) & 0 & \ldots & 0 & K_{N}(\beta r_{d})\\ \beta H_{-N}^{(1)\prime }(\beta r_{d}) & 0 & \ldots & 0 & \beta K_{-N}^{\prime }(\beta r_{d}) & 0 & \ldots & 0\\ 0 & \ddots & & \vdots & 0 & \ddots & & \vdots \\ \vdots & & \ddots & 0 & \vdots & & \ddots & 0\\ 0 & \ldots & 0 & \beta H_{N}^{(1)\prime }(\beta r_{d}) & 0 & \ldots & 0 & \beta K_{N}^{\prime }(\beta r_{d}) \end{array}\right],$$

s is the column vector with scattering coefficients $E_{n}\text{ and }F_{n}$ to be found, and c is the column vector with the complex Fourier coefficients already determined.

**Figure 4.**
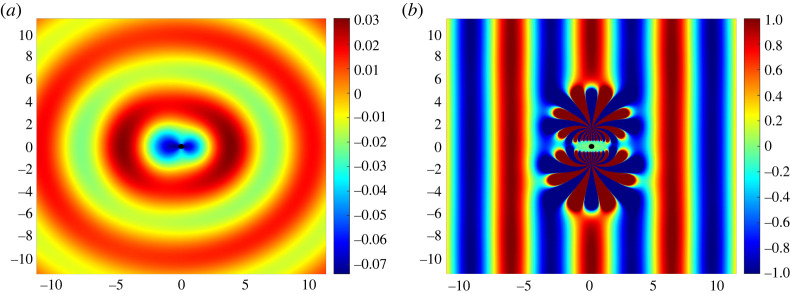
(*a*) The real part of the scattered field $u_{\text{sca}}$ due to the combined field $u_{\text{com}}=u_{\text{dev}}+u_{\text{inc}}$ in the ‘still’ region. The circular inclusion of radius $r_{d}=0.2$ is coloured black and positioned with its centre at the origin. (*b*) The real part of the total field $u_{\text{tot}}=u_{\text{com}}+u_{\text{sca}}$ generated by two devices positioned with up–down symmetry. The elliptical region and other parameter values are exactly the same as in figure 3*a*. (Online version in colour.)

In figure 4*a* we plot the scattered field $u_{\text{sca}}$ given in equation (4.2) for an inclusion of radius $r_{d}=0.2$ with its centre positioned at the origin, and in figure 4*b* we plot the total field $u_{\text{tot}}$ which encompasses the incident and scattered fields as well as the fields generated by the multipole devices. We note that, as expected, the inclusion lies entirely inside the elliptical region Ω. It is visually clear from a comparison of figure 1*a* and figure 4*b* that even two active evanescent devices are perfectly capable of cloaking the inclusion when the non-zero scattering is taken into account (also see the configuration with three devices presented in figure 1*b*).

## 5. Conclusion

Our emphasis is on the adoption of evanescent multipole devices as the foundation for an active cloak in a thin plate. This novel idea, exclusive to the biharmonic equation, exploits the rapidly decaying nature of the Macdonald functions $K_{n}$ to simplify the treatment of what is otherwise a difficult cloaking problem. In this introductory paper, we establish the underlying concept and demonstrate that as few as two multipole devices can successfully cloak and shield a clamped inclusion. We achieve an incremental improvement with three multipole devices and present a comparison between the two configurations. In fact, our results follow as a development from initial simulations in §3 that display a region of ‘stillness’ in the absence of an inclusion.

Immediate future work will consist of a rigorous approach to understanding the operation of the evanescent devices and their applications in different cloaking problems.

## Data Availability

This article does not contain any additional data.

## References

[1] Dolin LS. 1961 To the possibility of comparison of three-dimensional electromagnetic systems with nonuniform anisotropic filling. Izv. Vyssh. Uchebn. Zaved. Radiofizika **4**, 964-967.

[2] Kildishev AV, Cai W, Chettiar UK, Shalaev VM. 2008 Transformation optics: approaching broadband electromagnetic cloaking. New J. Phys. **10**, 115029. (10.1088/1367-2630/10/11/115029)

[3] Greenleaf A, Lassas M, Uhlmann G. 2003 Anisotropic conductivities that cannot be detected by EIT. Physiol. Meas. **24**, 413. (10.1088/0967-3334/24/2/353) 12812426

[4] Leonhardt U. 2006 Optical conformal mapping. Science **312**, 1777-1780. (10.1126/science.1126493) 16728596

[5] Pendry JB, Schurig D, Smith DR. 2006 Controlling electromagnetic fields. Science **312**, 1780-1782. (10.1126/science.1125907) 16728597

[6] Alù A, Engheta N. 2005 Achieving transparency with plasmonic and metamaterial coatings. Phys. Rev. E **72**, 016623. (10.1103/PhysRevE.72.016623) 16090123

[7] Mansfield EH. 1953 Neutral holes in plane sheet—reinforced holes which are elastically equivalent to the uncut sheet. Q. J. Mech. Appl. Math. **6**, 370-378. (10.1093/qjmam/6.3.370)

[8] Vasquez FG, Milton GW, Onofrei D. 2009 Broadband exterior cloaking. Opt. Express **17**, 14 800-14 805. (10.1364/OE.17.014800) 19687958

[9] Vasquez FG, Milton GW, Onofrei D. 2009 Active exterior cloaking for the 2D Laplace and Helmholtz equations. Phys. Rev. Lett. **103**, 073901. (10.1103/PhysRevLett.103.073901) 19792644

[10] Vasquez FG, Milton GW, Onofrei D. 2011 Exterior cloaking with active sources in two dimensional acoustics. Wave Motion **48**, 515-524. (10.1016/j.wavemoti.2011.03.005)

[11] Guevara Vasquez F, Milton GW, Onofrei D, Seppecher P. 2013 Transformation elastodynamics and active exterior acoustic cloaking. In *Acoustic metamaterials: negative refraction, imaging, lensing and cloaking* (eds RV Craster, S Guenneau), pp. 289–318. New York, NY: Springer.

[12] Norris AN, Amirkulova FA, Parnell WJ. 2012 Source amplitudes for active exterior cloaking. Inverse Prob. **28**, 105002. (10.1088/0266-5611/28/10/105002)

[13] O’Neill J, Selsil Ö, McPhedran RC, Movchan AB, Movchan NV. 2015 Active cloaking of inclusions for flexural waves in thin elastic plates. Q. J. Mech. Appl. Math. **68**, 263-288. (10.1093/qjmam/hbv007)

[14] Futhazar G, Parnell WJ, Norris AN. 2015 Active cloaking of flexural waves in thin plates. J. Sound Vib. **356**, 1-19. (10.1016/j.jsv.2015.06.023)

[15] O’Neill J, Selsil Ö, McPhedran RC, Movchan AB, Movchan NV, Moggach CH. 2016 Active cloaking of resonant coated inclusions for waves in membranes and Kirchhoff plates. Q. J. Mech. Appl. Math. **69**, 115-159. (10.1093/qjmam/hbw001)

[16] O’Neill J, Selsil Ö, Haslinger SG, Movchan NV, Craster RV. 2017 Active cloaking for finite clusters of pins in Kirchhoff plates. SIAM J. Appl. Math. **77**, 1115-1135. (10.1137/16M1088909)

[17] Abramowitz M, Stegun IA. 1965 Modified Bessel functions *I* and *K.* In *Handbook of mathematical functions with formulas, graphs, and mathematical tables*, pp. 374–377. New York, NY: Dover Publications.

[18] Banerjee S, Roy A. 2014 Linear algebra and matrix analysis for statistics. Texts in Statistical Science, vol. 181. Boca Raton, FL: CRC Press.

[19] Jones IS, Brun M, Movchan NV, Movchan AB. 2015 Singular perturbations and cloaking illusions for elastic waves in membranes and Kirchhoff plates. Int. J. Solids Struct. **69**, 498-506. (10.1016/j.ijsolstr.2015.05.001)

